# Improvement of Cr-Co-Mo Membrane Surface Used as Barrier for Bone Regeneration through UV Photofunctionalization: An In Vitro Study

**DOI:** 10.3390/ma10070825

**Published:** 2017-07-21

**Authors:** Oscar Decco, Jésica Zuchuat, Nicolás Farkas

**Affiliations:** Bioimplants Laboratory, Faculty of Engineering, National University of Entre Rios, Oro Verde, Entre Rios 3100, Argentina; odecco@bioingenieria.edu.ar (O.D.); nfarkas@bioimplantes.com.ar (N.F.)

**Keywords:** Cr-Co-Mo, Barriers, hydrophilicity, contact angle, photofunctionalization

## Abstract

Although there are several studies of the ultraviolet (UV) light-mediated photofunctionalization of titanium for use as implant material, the underlying mechanism is not fully understood. However, the results of in vitro and in vivo studies are very encouraging. The use of UV photofunctionalization as a surface treatment on other implant materials, as the Cr-Co-Mo alloy, has not been explored in depth. Using sandblasted Cr-Co-Mo discs, the surface photofunctionalization was studied for ultraviolet A (UVA, 365 nm) and ultraviolet C (UVC, 254 nm), and the surfaces were evaluated for their ability to sustain hydroxyapatite crystal growth through incubation in simulated body fluid for a seven-day period. The variation of the pre- and post-irradiation contact angle and surface composition was determined through the quantification of the weight percentage of Ca and P crystals by the EDAX ZAF method (EDS). Statistically significant differences (*p* < 0.05) were found for samples irradiated with UVA over 48 h, corresponding with hydrophilic surfaces, and the same result was found for samples exposed to 3 h of UVC. Superhydrophilic surfaces were found in samples irradiated for 12, 24 and 48 h with UVC. The decrease in the carbon content is related with the increase in the surface content of Ca and P, and vice versa over the Cr-Co-Mo surfaces.

## 1. Introduction

Implantable metallic biomaterials are non-living, used in a medical environment and designed to interact with biological systems [1]. Most importantly, the material must be biocompatible, which means that its presence within biological tissues does not cause harmful biochemical responses for those tissues or for the good performance of the substitution or repair functions [1]. As an additional characteristic, corrosion resistance must be mentioned, because if the metallic materials are oxidized in body fluid, toxicity reactions and in vivo allergy are promoted, leading to the release of metallic ions for an extended period of time and their combination with the body's biomolecules as proteins and enzymes [2]. In material science and engineering, studies have been focused on the behaviour, analysis and improvements of implantable materials’ properties with the aim of favouring their interaction with biological media, their biomechanical characteristics in terms of the applied loads on the structure replaced and their behaviour in a living organism.

In the area of implantology, the study of the design of dental implants and other implantable devices as well as the surface treatments and/or the different coatings to give adequate physicochemical properties to obtain a desired biological response continues to be the subject of research. In particular, metallic materials such as titanium (the gold standard) and the Cr-Co-Mo alloy have been two of the most studied [3,4,5,6]. Cr-Co alloys have been used in a wide variety of biomedical applications, including dental implants and as parts of orthopaedic prostheses [7,8]. The alloy shows high biocompatibility levels and has excellent properties including pitting, wear, abrasion and cracking-corrosion resistance, as well as a high fatigue resistance, malleability and ductility [9,10], although there is evidence that suggests toxicity reactions due to ion release in applications that include friction between contact surfaces of prosthetic devices [11,12]. Previous studies done by this research group [13,14] have shown that Cr-Co-Mo membranes provide an adequate space to allow bone ingrowth and its combination with both whole blood and platelet-rich plasma as growth promoters showed good results in bone height, volume and quality. Additionally, it has been observed that the membrane implantation itself, without the addition of factors or cells, possibly has promoted and originated proliferation, differentiation and cell adhesion, resulting in a consequent bone augmentation, similar to that demonstrated by Mustafa et al. (2001) [15] and Jayaraman et al. (2004) [16] for the titanium.

The material surface characteristics, composition and porosity are some of the properties that can be modified to improve the way the implanted material will relate to the host tissue and its response in the biological environment [17]. Wettability refers to the property of a surface that causes a liquid on the surface to tend to minimize or maximize the contact area between the liquid and the surface. When placing an implantable device into the maxillary bone and upon entering in contact with the blood, the interaction between the implant material surface and the wetness of the mouth determine how it will relate to blood, so this phenomenon determines the osteoconductive characteristic of the biomaterial. As a first approximation to determine the affinity between blood and a surface-treated biomaterial in a biological medium, methods to quantify wettability through the contact angle have been developed [18]. This magnitude of this simple and macroscopically observable measurement shows the material’s affinity for liquid. Water is generally used as a fluid medium, and these results are similar to those found for blood [19,20].

The biomaterial characteristics, storage time and storage medium influence its response to the biological environment. Ageing is the phenomenon whereby a metallic surface is contaminated by organic impurities, comprising principally hydrocarbons from the environment (atmosphere, water and other solutions) that promote the increase in the surface hydrophobicity. It has been demonstrated that the presence of these impurities decrease the affinity between water or blood and the material, with the consequent impoverishment of interaction between the phases [21]. Such contamination is proportional to time and greater when the material is less protected from the environment [22,23,24]. Aita et al. (2009) [24] have proposed a method for their elimination through ultraviolet (UV) light irradiation before the surgical implantation procedure.

UV photofunctionalization is an emerging technology in the biomedical field. According to Ogawa (2014) [25], a series of physicochemical alterations that occur in titanium improves the biological response in a series of in vitro studies, demonstrating a considerable improvement in the attachment, retention, and subsequent functional cascades of osteogenic cells derived from animals and humans after UV irradiation [26,27]. In the same way, in vivo studies found that bone morphogenesis induced around the UV-treated implants led to an improvement in the bone-implant osseointegration [24,28]. This treatment is simple, effective and applicable to all surface topographies commonly used in implantology [23,24,28]. UV photofunctionalization has been widely studied on titanium; however, recent studies on other materials such as Cr-Co-based alloys [29] and zirconia [30] have been developed.

The aim of the present work was to study the response in the surface of Cr-Co-Mo membranes after its UV photofunctionalization to improve their characteristics in relation to the hydrophilicity. An in vitro study was performed with Revised Simulated Body Fluid (R-SBF) to assess the UV-treated surfaces and deposition of apatite crystals over the surfaces, which should indicate that the material surface is adequate to the formation of a bone-like apatite layer.

## 2. Results

### 2.1. Contact Angle

To quantify the degree of affinity for the liquid medium, the physical principle described by Young and Dupré [31] was used. They formulated the mathematical equation that relates the surface tensions of a three-phase balance, in our case: solid, liquid and gas, considering the balance condition of the system through which is defined the balance contact angle of an ideal and hypothetic case. Tadmor (2004) [32] demonstrated that it is possible to calculate the balance angle defined by the Young–Dupré equation depending on the maximum values of advancing and receding angles. In this work, we calculated the balance contact angle according to the Tadmor equation [32] (Equation (1)).

(1) $$\theta_{0}=arccos\left(\frac{r_{a}cos\theta_{a}+r_{r}cos\theta_{r}}{r_{a}+r_{r}}\right),$$

where

(2) $$r_{a}={\left(\frac{{sin}^{3}\theta_{a}}{2-3cos\theta_{a}+{cos}^{3}\theta_{a}}\right)}^{1/3},$$

and

(3) $$r_{r}={\left(\frac{{sin}^{3}\theta_{r}}{2-3cos\theta_{r}+{cos}^{3}\theta_{r}}\right)}^{1/3},$$

$$\theta_{0}=Balance\;contact\;angle$$

$$\theta_{a}=Advancing\;contact\;angle$$

$$\theta_{r}=Receding\;contact\;angle$$

Mean values (±standard deviation (SD)) of balance contact angles before and after UV irradiation are shown in Table 1. To determine any significant difference between the groups, data obtained were subjected to a one-way analysis of variance (ANOVA) and Tukey’s honest significant difference (HSD) test; the significance was determined at the 95% confidence level. The analysis of contact angle values before irradiation show statistically significant differences (*p* < 0.05) between the contact angle values for all groups tested.

**Figure 1:**
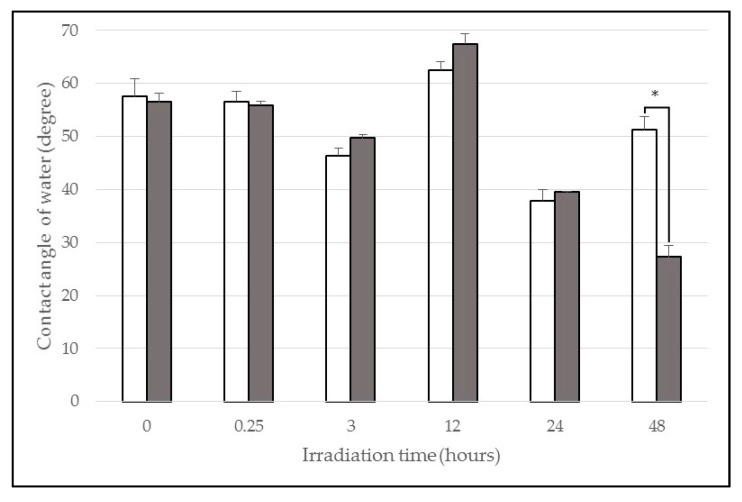
Contact angle variation of UVA irradiation. There were no statistically significant differences for the first four irradiation times studied. * For 48 h of UVA irradiation statistically significant difference (*p* < 0.05) was observed.

To statistically analyse each wavelength as a function of irradiation time, it was demonstrated that, for samples irradiated with 365 nm, no statistically significant differences between contact angles for the groups corresponding to 0.25, 3, 12 and 24 h were found; sometimes, an increase in the values of the angles after irradiation was observed (Figure 1). However, for the group of samples irradiated for 48 h, statistically significant differences (*p* < 0.05) were observed, corresponding with a decrease in the values after irradiation, resulting in hydrophilic surfaces.

**Figure 2:**
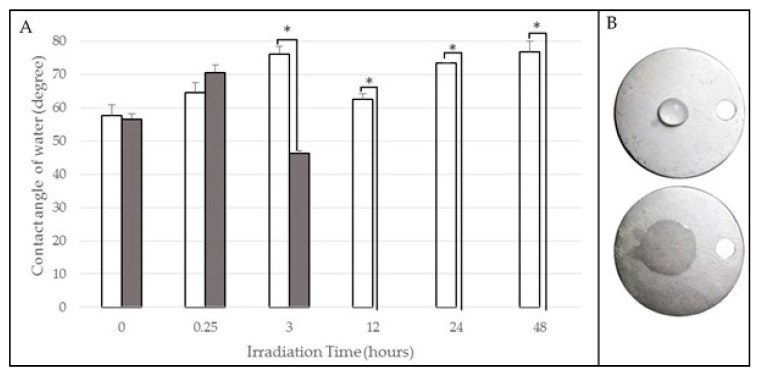
(**A**) Contact angle variation of UVC irradiation. There were no statistically significant differences for 15 min of irradiation. After 3 h, statistically significant differences were observed: * (*p* < 0.05), and from 12 h, superhydrophilic surfaces were found; (**B**) The control (upper) and the 48 h discs (lower) after UVC irradiation.

For the samples irradiated with 254 nm, there were no statistically significant differences between the values for pre- and post-irradiation contact angles for the group exposed for 15 min. However, for the subsequent irradiation times, statistically significant differences (*p* < 0.05) were found between the angles, before and after irradiation, from 3 h (Figure 2), resulting in superhydrophilic surfaces from 12 to 48 h of irradiation. To analyse the values for the control group (non-irradiated samples), there were no statistically significant differences between both measurement times (Figure 1 and Figure 2).

### 2.2. In Vitro Culture

**Figure 3:**
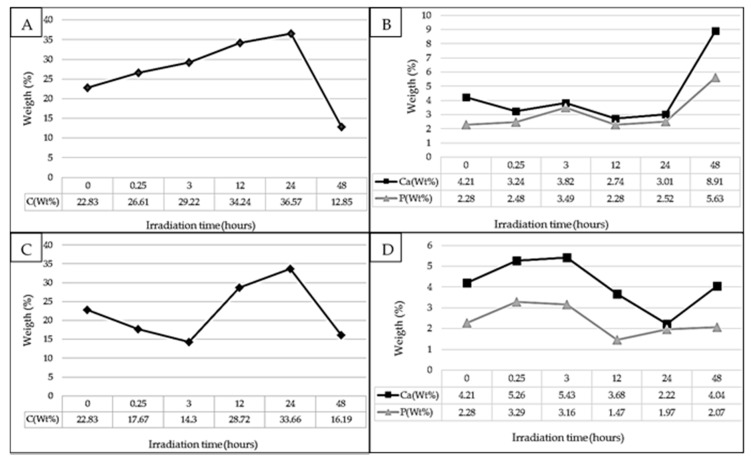
Microanalysis of surfaces irradiated. (**A**,**C**) Carbon content (UVA and UVC, respectively); (**B**,**D**) Ca and P content (UVA and UVC, respectively). High-resolution figure.

The disc surface composition after culture was analysed through EDS (Energy Dispersive Spectroscopy) by the standard-less EDAX ZAF quantification method. The weight percentage of apatite crystals (Ca and P), the alloy elements (chromium, cobalt and molybdenum) and carbon were quantified to determine, on the one hand, the new membrane surface ability to sustain hydroxyapatite crystal growth and, on the other hand, to evaluate the surface contamination during their manipulation and storage until the microanalysis or another possible contamination during the analysis in the SEM (Scanning Electron Microscopy) by deposition of hydrocarbons from the chamber vacuum. The proportion of elements found in the mapping of the surface of samples irradiated with UVA are shown in Figure 3A,B, where it can be seen how the irradiation time does not alter the surface content of carbon; but it increases with the increase of the irradiation time. In addition, no significant differences in the presence of calcium and phosphorus are observed until 24 h.

Similar to the findings for the contact angle, a significant decrease in the content of carbon was observed in the sample following 48 h of irradiation. This correlates with an increase in the deposition of apatite crystals on the surface. An initial decrease in the content of carbon is observed (until 3 h of irradiation) in the samples irradiated with UVC with respect to the control (non-irradiated) and then it increases again to even higher values (with respect to the initial values) to the samples corresponding to 12 h and 24 h of treatment (Figure 3C,D). Equivalent to that observed with UVA radiation, the content of carbon abruptly decreases for discs irradiated during 48 h of treatment. The percentage concentration of apatite crystals are correlated with the carbon content: decreasing carbon, increases the apatite crystals, and vice versa. The average ratio of Ca/P in terms of atomic number (%) was 1.06 to samples irradiated with UVA; and 1.38 to samples irradiated with UVC. Human hydroxyapatite ratio Ca/P is 1.67.

**Figure 4:**
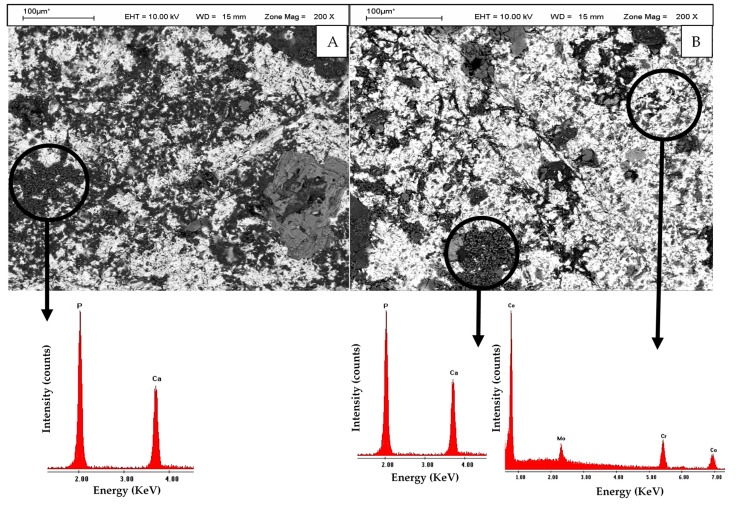
Micrograph and Energy Dispersive Spectroscopy (EDS) spectra of samples of Cr-Co-Mo alloy irradiated with UV light during 3 h, incubated in Revised Simulated Body Fluid (R-SBF) for seven days. (**A**) UVA-treated; (**B**) UVC-treated.

**Figure 5:**
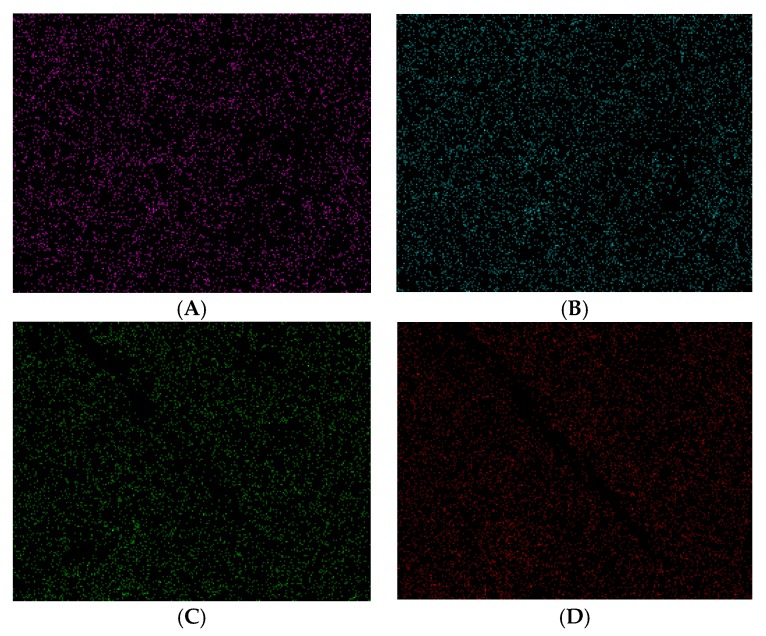
Mapping of surface Ca (**A**) and P (**B**) distribution to the 3 h UVA-irradiated samples, and superficial Ca (**C**) and P (**D**) to its homologous sample, UVC-irradiated.

In Figure 4B, there is a micrograph and EDS spectra corresponding to the sample of 3 h of exposure to UVC where the greatest removal of surface carbon was observed, which corresponds to a Ca/P content ratio similar to that presented by the human hydroxyapatite. The same graphs, corresponding to the samples of 3 h UVA exposure, are shown in Figure 4A. The surface distribution of Ca and P crystals on the area of study (for both wavelengths) is shown in Figure 5.

## 3. Discussion

The major source of implant contamination during the fabrication process occurs within the first few seconds of exposure to oxygen in the air, contaminating the surface by hydrocarbon deposition, which translates into a decrease of surface free energy that is subsequently reflected in an increase in the contact angle [33,34]. Previous studies have informed that progressive deposition of hydrocarbons onto titanium surfaces is inevitable in the practice of medicine [35,36]. Morra et al. (2003) [37] analysed the chemical composition of several types of implant surfaces, showing a carbon deposition of 17.9–76.5% in 34 titanium dental implants tested independently of topography, suggesting that hydrocarbon deposition may occur on titanium-based materials with any surface texture. Although it has been extensively investigated with titanium, all implants used today have a certain hydrocarbon contamination binding to the surface. Unsaturated bonds on their surface are saturated by the adsorption of contaminant molecules, which are always present in small amounts in the air [38].

To evaluate the hydrocarbon contamination and to demonstrate the effectiveness of the effects of the surface treatment on the implant materials, contact angle measurement as a method to characterize the new material surfaces in a reliable way is used [39]. This parameter is useful to assess the macroscopic surface properties, such as the surface energy and wettability, indicating the effectiveness of the treatment for increasing hydrophilicity, which will correspond to a higher affinity between the blood and the implant material in its clinical application [40,41].

In our research, Cr-Co-Mo discs with the same surface treatments as described by Alfarsi et al. (2014) [33] were used as a representative sample of implants. They were allowed to age up to eight weeks before UV treatment, with the aim of verifying the hydrocarbon deposit on the surface and to check their influence in the contact angle values to evaluate the change in the hydrophilicity after their exposure to UV light, quantify the contact angle of each situation and to statistically analyse the results. In this respect, statistically significant differences were found (*p* < 0.05) between the values before irradiation of all the samples, although they had been stored individually in Petri’s capsules under the same conditions, so it is postulated that the hydrocarbon deposition on the disc surfaces is produced in a random way and is highly dependent of the composition of the encapsulated air. Relatively large standard deviations (ranged from 0.1 to 3.37, even normally distributed according to Shapiro–Wilk test) in the measurement of contact angles for the same disc, in successive iterations could indicate that the image capture and therefore the subsequent measurement of contact angle is altered by the atmospheric conditions during its manipulation and drying within an air circulation chamber, which can be sources of hydrocarbon contamination.

Samples were irradiated with UV light at 365 nm and 254 nm for the determined period according to the protocol described in Section 4.2. After UVA photofunctionalization, the results did not show significant differences for all the irradiation times under 48 h (Figure 1), unlike UVC-irradiated samples, for which statistically significant differences were found for all irradiation times after 3 h (Figure 2). These results could be argued by analysing the intrinsic properties of each wavelength, since UVC radiation of *λ* = 254 nm (corresponding to the interval of maximum ozone absorption) promotes, in a direct way, the simultaneous formation and decomposition of ozone and it is capable of breaking up high-energy chemical bonds, as the ethylenic carbon-carbon double bond [42], removing impurities present in the surface. Statistically significant differences (*p* < 0.05) at 3 h after UVC irradiation were observed with respect to pre-irradiated angles, which correspond to hydrophilic surfaces. From 12 h, superhydrophilic surfaces were found. These results correspond with those found by Aita et al. (2009) [24,26], who exposed their titanium samples to 48 h of UVC irradiation (intensities of approximately 2 mW·cm^−2^). The same way, Al Qahtani et al. (2015) [21] found similar results at 40 min of exposure using radiation sources with intensities of approximately 15 mW·cm^−2^ in comparison to 2 mW·cm^−2^ used in our work. Att et al. (2009) [23] have exposed a Cr-Co alloy to UVC radiation during 48 h, finding superhydrophilicity; the same results were obtained in our study from 12 h of exposure.

There were no statistically significant differences between contact angle values before and after the treatment for any of the samples exposed for 15 min to both wavelengths. Samples irradiated with UVA for 48 h showed statistically significant differences (*p* < 0.05), corresponding with hydrophilic surfaces, which is related to a decrease in the content of surface carbon, according with results from microanalysis (Figure 3A). While the decrease of carbon is achieved, the mechanism through which it is carried out would be by photocatalysis, similar to that found by Zubkov et al. (2005) [43] for titanium.

Immediately after UV radiation, samples were incubated in vitro for seven days at 37 °C to evaluate and quantify the deposition of apatite crystals on the surfaces treated with UVA and UVC, similar to what was done by Kokubo et al. (1996) [44], who demonstrated that the essential requirement for an implant to bond to the living bone is the formation of a bone-like apatite layer. The formation of this layer in the implant surfaces can be reproduced under experimental conditions by immersing these materials in acellular simulated body fluid (SBF), with ion concentrations (Cl^−^ and HCO_3_^−^) nearly equal to those of human blood plasma. Bone-like apatite layer formation on the material surface is useful for the in vitro investigation of bioactivity and biomimetic synthesis of apatite layer since it played crucial roles in osteoinduction. In this case, we used the new formulation (R-SBF) proposed by Kim et al. (2001) [45], corresponding to the revised version, which is actually used in most of the in vitro studies. As reported by Att et al. (2009) [23], the bioactivity of Ti surfaces was demonstrated by the higher attachment and cell density of osteoblasts for 48 h of UVC-irradiated samples and incubated for 24 h with respect to the results for 3 h of incubation.

To samples irradiated with UVA, the microanalysis results show that after culture, the surface carbon content is increased in all the samples irradiated up to 24 h (Figure 3A), corresponding with the decrease in the weight percentage of calcium and phosphorus (Figure 3B), differing from that observed at the sample of 48 h where the opposite was observed. To discs irradiated with UVC, a decrease in the carbon content was observed for samples irradiated for 15 min, 3 h and 48 h (Figure 3C), with the increase of proportion of Ca and P (Figure 3D); a marked increase in the content of carbon to the samples irradiated for 12 and 24 h, corresponding with a decrease in the content of Ca and P. This difference in the element quantification could be possible due to the small size of the mapping area (1 mm^2^) in relation with the disc surface. In addition, the distribution of crystals is not uniform (Figure 4 and Figure 5), but varies depending on the place where the sample is focused to analyse it through EDS; in addition to the organic contaminants that can be deposited during the manipulation and storage of the samples or during the analysis in the SEM. In this sense, we must mention the importance of the elapsed time between the finishing of the culture and the quantitative X-ray microanalysis. According to Att et al. (2009) [23], the atomic percentage of carbon on titanium surface increases from 20% to 60% in four weeks in a regular atmosphere; and, according to Denzer et al. (2002) [42], the low-carbon state (8–13%) is practically maintained without changes for at least one day. When comparing the average of the Ca/P ratio of samples irradiated with each wavelength, it was observed that the UVC-treated samples present a value close to that of human hydroxyapatite, while the average of the Ca/P ratio of UVA-irradiated samples was lower.

In concordance with conclusions reported by Aita et al. (2009) [24,26] and Hori et al. (2010) [27], some of the results found in the present study showed that a decrease in the contact angle may not indicate a higher deposition of Ca and P crystals over the surface. It suggests that incongruences can arise when relating the results of contact angle and surface bioactivity, suggesting the existence of a series of variables that could be affected by UV radiation, but at the moment they have not been identified, registered or controlled, which makes impossible an analysis of the relationship between the registered variables, and any conclusions based on them. Beyond this, in clinical practice, the surface superhydrophilic behaviour could favour the blood–implant contact, speeding up the cicatrization process [21].

## 4. Materials and Methods

### 4.1. Cr-Co-Mo Samples and Surface Characterization

To determine and quantify changes in hydrophilicity caused by the surface treatment, Cr-Co-Mo (ASTM F75) discs (2 mm thick, 20 mm diameter) were prepared by casting using the lost-wax technique. The discs were subjected to a series of processes of cut and polish to obtain samples relatively free of surface defects (whose generation is intrinsic to the casting). After that, surfaces were sandblasted with 220 μm aluminium oxide powder and the discs were washed in ultrasound bath with bi-distilled water for 10 min to remove the waste from the surface treatment. Samples were left to age for 2 months and stored individually in sterile containers in a dry and dark environment at 21.85 °C average temperature, 77.48% average relative humidity. The geometry of the samples to in vitro testing was in accordance with Alfarsi et al. (2014) [33], who used discs with surfaces equivalent to the dental implant surface currently used in clinical practice.

### 4.2. Ultraviolet-Light Irradiation

The irradiation protocol included two wavelengths of ultraviolet spectrum: 254 nm (UV-C) and 365 nm (UV-A). The two ultraviolet light sources, 15 W tubes, intensity approximately 2 mW·cm^−2^, were positioned at 3 cm above the discs. Five discs were treated with each wavelength for different periods of times: 15 min, 3, 12, 24 and 48 h. A non-irradiated sample was kept as control.

### 4.3. Contact Angle Measurement 

**Figure 6:**
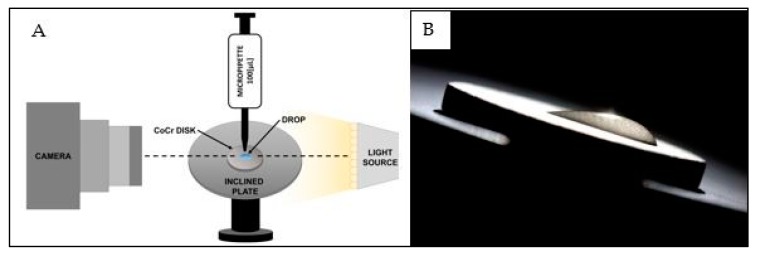
(**A**) Setup for image capture; (**B**) Image used for measurement.

The setup, on which samples were deposited to the corresponding measurements, comprised a base with a surface slope of 45°, experimentally calculated according to the macroscopic slip-limit of the drop (Figure 6). A Reflex Digital Camera (Canon Eos KissX5^®^, Canon, Tokyo, Japan), F point 5.6, focal distance of 60 mm, exposition time of 1/100 s, ISO 100, Canon, Tokyo, Japan) supplied with macro lens (Canon^®^ EF-S 60 mm f/2.8), was fixed perpendicularly to the sample plane and placed on a tripod. LED retro-illumination was used to maximize the contrast between the material and the drop of water.

**Figure 7:**
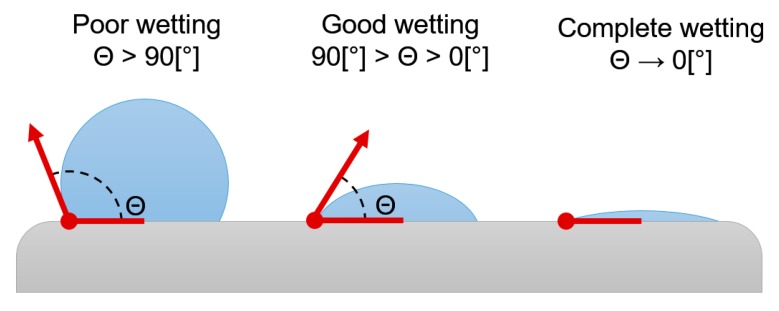
Characterization of contact angle.

Protocol for pre- and post-irradiation image capture consisted on the deposition of a 100 µL drop of bi-distilled water using a single-channel pipettor (DragonLab^®^, Beijin, China), and the image capture. All steps in this procedure have been repeated at least three times to each sample, prior to drying within an air circulation chamber. After the image capture, from which the initial contact angles were quantified for each group, the samples were irradiated according to the protocol described in the previous section. Once the irradiation time has been reached, the discs were extracted and post-irradiation images were captured. Finally, the samples were stored individually in Petri’s capsules. Advancing and receding contact angles corresponding to the pre- and post-irradiation states, based on which balance angle was calculated, were quantified by an image analyser (ImageJ, NIH, Bethesda, ML, USA) and characterized as shown in Figure 7.

### 4.4. Immersion in R-SBF

After the irradiation protocol and immediately after the post-irradiation contact angle measurement, the samples were kept individually in plastic containers where they were covered with R-SBF [45] according to Kokubo et al. (2006) [46] and incubated in vitro for 7 days. After this period, the solution was removed; discs were dried in an air circulation chamber and kept individually until their analysis. Surface topography analysis and mapping was carried out using scanning electron microscopy (SEM, LEO 1450VP, LEO Electron Micros-copy, Ltd., Cambridge, UK) with energy dispersive X-ray spectroscopy (EDS, Genesis 2000 XMS, EDAX Inc., Mahwah, NJ, USA).

### 4.5. Statistical Analysis

The number of samples was 3 for all the studies (*n* = 3). SPSS^®^ (Chicago, IL, USA) statistical software was used and one-way analysis of variance (ANOVA) was employed to determine the difference among groups. Those values of *p* < 0.05 were considered significant.

## 5. Conclusions

It was found that, for Cr-Co-Mo sandblasted surfaces, physicochemical modifications mediated by UV were verified, similar to that found in titanium. Hydrophilic surfaces with good wetting properties were found at 3 h of UVC irradiation and superhydrophilic surfaces after 12 h. After 48 h of UVA irradiation, hydrophilicity was found in the surface of Cr-Co-Mo discs, which may indicate that for higher exposition times, the wetting of the surface could improve. However, more studies with irradiation periods longer than 48 h should be done, considering that UVA radiation implicates less risks than UVC, which justifies its use if comparable results are obtained, although they would be for higher treatment times. It was established that the decrease in the content of carbon is related with the increase in the surface content of Ca and P, and vice versa. Additionally, the average Ca/P ratio is closer to the ratio in the human hydroxyapatite in samples irradiated with UVC and incubated during seven days in comparison with UVA-irradiated and subjected to the same study.

## Figures and Tables

**Table 1:** Contact angle values before and after irradiation.

Wavelength (nm)	Irradiation Time (h)	Contact Angle Pre-Irradiation (°)	Contact Angle Post-Irradiation (°)
254 (UVC)	0.25	65 ± 3	71 ± 2
	3	76 ± 2	46 ± 1
	12	62 ± 2	0 ± 0
	24	73 ± 0	0 ± 0
	48	77 ± 3	0 ± 0
365 (UVA)	0.25	55 ± 2	56 ± 1
	3	46 ± 1	50 ± 1
	12	63 ± 2	67 ± 2
	24	38 ± 2	39 ± 0
	48	51 ± 2	27 ± 2
Control	Non-irradiated	58 ± 3	57 ± 2
